# Thoracolumbar Chance fracture during a professional female soccer game: case report

**DOI:** 10.1590/S1679-45082016RC3432

**Published:** 2016

**Authors:** Alberto Ofenhejm Gotfryd, Fernando José Franzin, Roger Hartl

**Affiliations:** 1 Hospital Israelita Albert Einstein, São Paulo, SP, Brazil.; 2 Weill Cornell Brain and Spine Center, New York, NY, United States.

**Keywords:** Spine/injuries, Fractures, bone, Athletes, Bone screws, Case reports

## Abstract

We report a rare case of an unstable flexion-distraction spine fracture with ligament involvement that occurred during a professional female soccer game. There were no neurological *déficit*. The patient had a painful midline gap which suggested ligamentar injury that was not immediately recognized. Despite that, proper immobilization and referral to hospital for further evaluation avoided additional spinal cord damage. The patient underwent a monosegmental posterior instrumentation spine fusion and after 6 months returned to professional soccer activities. This paper alerts to the possibility of occurrence of severe and unstable spine injuries during soccer practice and the importance of an adequate initial care at the game field in order to avoid iatrogenic neurological injuries.

## INTRODUCTION

Spine fractures may occur in collision athletes. In this population, most fractures are compressive, primarily affect the cervical spine and are mechanically stable. Flexion-distraction injuries, also known as Chance fractures, are classically caused by high-energy traumas, such as motor vehicle accidents and falls. These fractures are potentially unstable and often related with neurological *déficits*.

## OBJECTIVE

To warn of the possibility of occurrence of unstable fractures during soccer practice and the management of these injuries in professional athletes.

## CASE REPORT

A 23 year-old female professional soccer player (JQC) had a spine trauma after colliding with an opponent player while jumping. She reported that her opponent jumped higher than her and fell down on her back, which forced her torso in flexion. Immediately after the trauma, the patient had intense pain in the back and was unable to continue playing.

**Figure 1:**
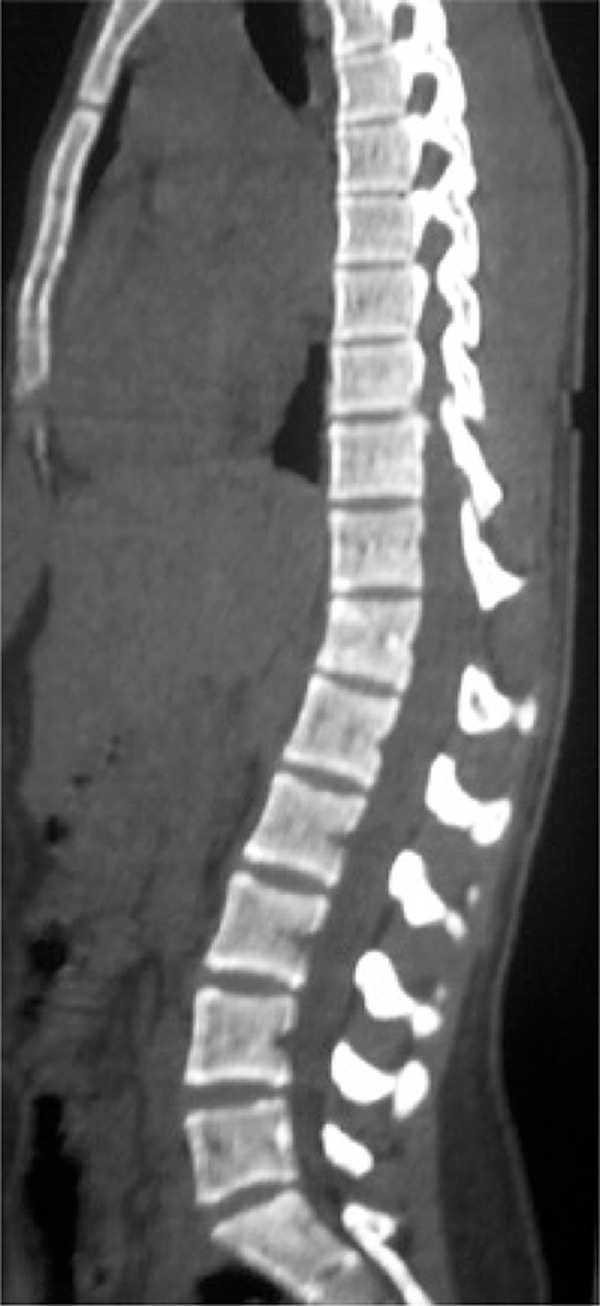
Sagittal computed tomography scan showing an enlargement of T11 and T12 spinous processes, which suggests a ligament injury

**Figure 2:**
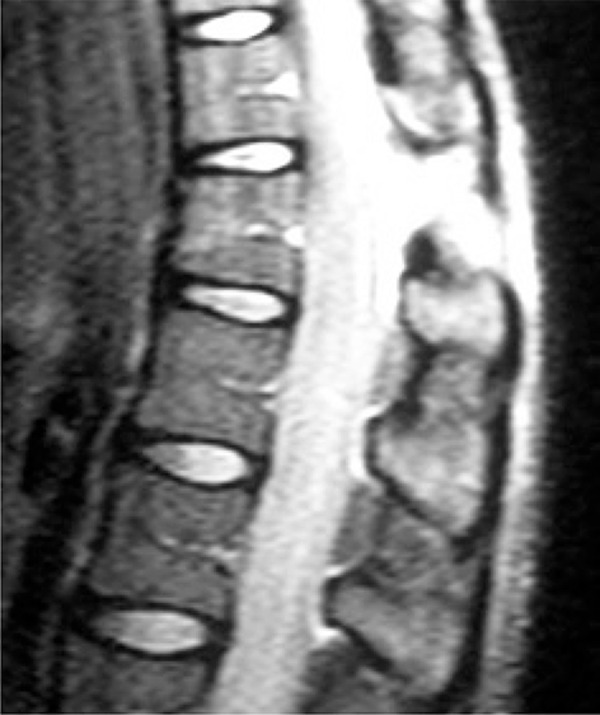
Magnetic resonance imaing showing complete rupture of posterior ligaments (supra and interspinous) between T11 and T12

There was no pain radiation to lower limbs, numbness or loss of strength. She was immobilized with cervical collar on a spine board and then referred for urgent medical evaluation at a nearby hospital. Physical examination revealed a dorsal midline painful gap and edema on the topography of thoracolumbar transition, but with no neurological impairment. Computed tomography scan revealed a T12 fracture and increased space between T11-T12 spinous processes (Figure 1). Because of the suspicion of a posterior ligament complex injury, a magnetic resonance imaging was done, and it revealed a complete T11-T12 posterior ligament injury (Figure 2).

The fracture was classified as AO B2^1^ type, that is mechanically unstable, and therefore surgical treatment was indicated. The patient was temporarily immobilized with a thoracic-lumbar-sacral orthosis and transferred to another city where the surgery was performed 3 days after the trauma. The patient underwent a posterior monosegmental fusion (T11-T12) with instrumentation called “interne fixator” (Depuy Synthes, United States) combined with autologous iliac graft.

## RESULTS

The patient was discharged 2 days after the procedure without external immobilization. Two weeks after the surgery, she initiated physical rehabilitation with exercises to maintain muscle tone, but she was partial restricted to perform rotational movements and bending of the trunk. As local pain decreased, speed walking, stationary biking and resistance-type weight lifting exercises for lower extremities were introduced. The exercise program had progressive intensity and last for 6 months.

**Figure 3:**
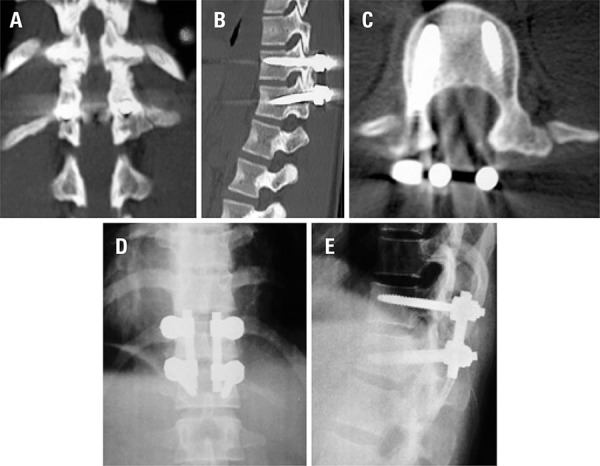
(A) coronal computed tomography scan showing complete T11-T12 fusion; (B) sagittal computed tomography scan showing complete T11-T12 fusion; (C) axial postoperative computed tomography scan; (D) postoperative anteroposterior X-ray showing proper coronal spine alignment; (E) postoperative lateral X-ray showing proper sagittal spine alignment

After six months, the patient was authorized to return to her professional activities without restrictions. In the 4-year follow-up after the surgery, she maintained a professional athlete’s routine without back pain or physical limitations. There were no complications related to implants (screw loosening, pull out or breakage) or radiographic signs of the adjacent level disease. The last follow-up images performed 4 years after the procedure showed solid bilateral bone fusion (Figures 3A to 3E).

## DISCUSSION

The occurrence of spine fractures during sports activities is quite frequent and they mainly involve the cervical spine.^2^Most of described cases were caused by compression mechanisms.^2^Boden et al.^3,4^ reviewed cases of severe spinal injuries during sports practice in the United States, and they concluded that soccer, ice hockey, wrestling, diving, snowboarding and rugby were the risky sports regarding spine lesions. In the United States, approximately 8.7% of all new cases of spinal cord injuries are related to sports practice.^3-5^

In 1948, Chance,^6^ described a spinal fracture characterized by a horizontal vertebral line affecting posterior elements (spinous process, lamina, transverse processes and pedicles) with extension to the vertebral body. At that time such injuries mainly occurred after car accidents, and especially in those who were using a “two points” seat belt. For this reason, the injury became popularly known as “seat belt fracture”. The Chance or seat belt fracture, as initially described, affects only bone elements. For this reason, it is likely to be treated conservatively with brace or hyperextension plaster cast.

However, when rupture of the posterior tension band complex is present, as observed in our case, surgical treatment is indicated especially because ligament healing is inadequate and instability may occur.^6^ These lesions are often described as Chance fracture with ligament involvement.

Chance fractures are caused by high-energy mechanisms, therefore, they are rarely described during sports practice. The only case reported of Chance fracture during sports activities was during a 12-foot high snowboarder fall.^7^However, there are several reports of thoracolumbar spine fractures (AO type A) during sports such as rugby, football, basketball, wrestling, winter sports and diving.^3-5^

A detailed physical examination may reveal dorsal pain, hematoma or painful gap. This latter is highly suggestive of posterior ligamentar injury. In this case report, such injury was not recognized during the initial approach at the game field. However, proper immobilization and referral to the hospital for further evaluation avoided additional spine damage.

The initial care of a patient with spine trauma includes proper immobilization of the affected segment, use of cervical collar and/or rigid board, and immediate orthopedic evaluation.^8^ The detection of interspinous space enlargement on X-rays or CT scan suggests flexion-distraction injuries. Such fractures may also have minimum compression of the anterior portion of the vertebral body.^6^ When posterior ligamentar injury is suspected, a magnetic resonance imaging better identifies it.^7^ In addition, the magnetic resonance imaging may provide important information about other soft structures, such as intervertebral discs, spinal cord and roots, specially when neurological disorders are present.

Treatment is defined based on the presence of mechanical instability or neurological impairment.^6-8^ Pure bone lesions, as described by Chance, can be treated with hyperextension orthosis, and often have a good clinical result.^6^ The presence of a complete ligament injury, as observed in our case, is an indication for spine stabilization in order to prevent further instability.^6,7,9^ As anterior elements are usually intact, there is no need for anterior column reconstruction.^6^

In our case, instrumentation was performed with AO internal fixator using Schanz screws (Synthes, United States). This system provides a significant lever arm to facilitate reduction of the fracture and restore the sagittal contour of the spine.^10^ An adequate spine fusion is expected to occur about 3 to 6 months after surgery and could be confirmed by computed tomography scan. The computed tomography scan performed 6 months after the surgery showed a solid T11-12 fusion. At that point, the rehabilitation program was intensified and restrictions were no longer necessary regarding sports activities.

## CONCLUSION

This paper alerts to the occurrence of severe spine lesions during soccer practice and to the importance of adequate initial care at the game field in order to avoid iatrogenic neurological lesions. If a complete posterior spine tension band injury is present, the injury is considered mechanically unstable and eligible to surgical treatment.
