# Supplementary material for: Thoracolumbar Chance fracture during a professional female soccer game: case report
Source: Einstein (Sao Paulo). 2016 Jan-Mar;14(1):67–70. doi: 10.1590/S1679-45082016RC3432 (PMC4872921; doi:10.1590/S1679-45082016RC3432)
Supplement: Translation

# Translation: Fratura toracolombar tipo Chance durante jogo de futebol feminino profissional: relato de caso

**Authors:** Alberto Ofenhejm Gotfryd, Fernando José Franzin, Roger Hartl

**Affiliations:** 1 Hospital Israelita Albert Einstein, São Paulo, SP, Brasil.; 2 Weill Cornell Brain and Spine Center, Nova Iorque, NY, Estados Unidos.

**Keywords:** Coluna vertebral/lesões, Fraturas ósseas, Atletas, Parafusos ósseos, Relatos de casos

## Abstract

Relatamos um caso raro de fratura instável da coluna vertebral com envolvimento ligamentar, ocorrida por mecanismo de flexão-distração, durante jogo de futebol feminino profissional. Não houve *déficit* neurológico. A paciente apresentava espaçamento doloroso dorsal na linha média, que sugeriu lesão ligamentar, a qual não foi reconhecida imediatamente. Apesar disso, realizaram-se imobilização adequada e encaminhamento para unidade hospitalar, fatos que evitaram a ocorrência de danos adicionais à medula espinal. A paciente foi submetida à fusão monossegmentar, com instrumentação e, após 6 meses, retornou à prática de futebol profissional. O presente estudo alerta para a possibilidade de ocorrência de lesões graves e instáveis na coluna durante a prática de futebol, e para importância da assistência inicial adequada ainda em campo, a fim de evitar lesões neurológicas iatrogênicas.

## INTRODUÇÃO

Fraturas da coluna vertebral podem ocorrer em atletas de colisão. Nessa população, a maioria das fraturas são por compressão e afetam principalmente a coluna cervical, além de serem mecanicamente estáveis. As lesões em flexão-distração, também conhecidas como fratura de Chance, são classicamente causadas por meio de traumas de alta energia, tais como acidentes automobilísticos e quedas. Tais fraturas são potencialmente instáveis e mais comumente relacionadas a *déficits* neurológicos.

## OBJETIVO

Relatar a possibilidade de ocorrência de fraturas instáveis durante prática de futebol e o tratamento dessas lesões em atletas profissionais.

## RELATO DE CASO

Paciente do sexo feminino, JQC, 23 anos de idade, jogadora profissional de futebol, sofreu trauma no dorso após colidir com jogadora oponente durante salto. A paciente relatou que sua oponente pulou mais alto do que ela e caiu sob suas costas, forçando-a a flexionar-se. Imediatamente após o trauma, a atleta apresentou dor intensa nessa região dorsal e não foi capaz de continuar no jogo.

Não foram relatadas dor irradiada para os membros inferiores, parestesia e nem perda de força. A paciente foi imobilizada com colar cervical e prancha longa, e, então, encaminhada para avaliação de urgência ao hospital mais próximo. Ao exame físico, observaram-se espaçamento doloroso dorsal na linha média e edema na topografia da transição toracolombar. Não havia alteração neurológica. A tomografia computadorizada revelou fratura em T12 e aumento do espaço entre os processos espinhosos de T11-T12 (Figura 1). Devido a suspeita de lesão do complexo ligamentar posterior, foi realizada ressonância magnética, que revelou lesão ligamentar posterior em T11-T12 (Figura 2).

**Figura 1:** Tomografia computorizada sagital mostrando alargamento dos processos espinhosos em T11 e T12, sugerindo lesão ligamentar

**Figura 2:** Ressonância magnética por imagem mostrando ruptura completa dos ligamentos posteriores (supra e interespinhosos) entre T11 e T12

A fratura foi classificada como tipo AO B2,^1^ ou seja mecanicamente instável, e houve indicação de tratamento cirúrgico. A paciente foi imobilizada temporariamente com órtese tóraco-lombo-sacra e transferida para outra cidade, onde foi submetida à cirurgia 3 dias mais tarde. Realizou-se fusão monossegmentar posterior (T11-T12), com instrumentação denominada “fixador interno” (Depuy Synthes, Estados Unidos), associada a enxertia óssea autóloga de crista ilíaca.

## RESULTADOS

A paciente recebeu alta hospitalar 2 dias após o procedimento sem imobilização externa. Duas semanas após a cirurgia, iniciou-se reabilitação física com exercícios para manter o tônus muscular, com restrição parcial de movimentos rotacionais e curvatura do tronco. Com a diminuição da dor, introduziram-se caminhadas, bicicleta ergométrica e exercícios de resistência dos membros inferiores com elevação de peso. O programa de exercícios progrediu em intensidade e teve duração de 6 meses.

Após 6 meses, a paciente foi autorizada a retornar suas atividades profissionais sem restrições. No seguimento de 4 anos após a cirurgia, mantinha a rotina de atleta profissional e não apresentava dores nas costas e nem limitações físicas. Não foram observadas complicações relacionadas aos implantes (frouxidão, soltura ou quebra de parafusos) ou sinais radiográficos de doença do nível adjacente. As imagens do último seguimento, realizadas 4 anos após o procedimento, mostraram fusão óssea bilateral sólida (Figuras 3A a 3E).

**Figura 3:** (A) tomografia computorizada coronal mostrando fusão completa de T11-12; (B) tomografia computadorizada sagital mostrando fusão completa em T11-T12; (C) tomografia computadorizada axial no pós-operatório; (D) raio X anteroposterior após a cirurgia, mostrando alinhamento coronal adequado da coluna; (E) raio X lateral após a cirurgia, mostrando alinhamento sagital adequado da coluna

## DISCUSSÃO

A ocorrência de fraturas da coluna vertebral durante atividades esportivas é relativamente frequente e afeta principalmente a coluna cervical.^2^ A maioria dos casos descritos foram causados por mecanismos de compressão.^2^ Boden et al.^3,4^ revisaram casos de lesão de coluna grave durante a prática esportiva nos Estados Unidos e concluíram que futebol, hóquei no gelo, luta olímpica, mergulho, *snowboard* e rúgbi foram os esportes com maiores risco em relação às lesões de coluna. Nos Estados Unidos, aproximadamente 8,7% de todas os novos casos de lesões da medula espinal são relacionadas à prática esportiva.^3-5^

Em 1948, Chance^6^ descreveu fratura da coluna vertebral caracterizada por linha horizontal vertebral afetando elementos posteriores (processo espinhoso, lâmina, processos transversos e pedículos), com extensão ao corpo vertebral. Naquele momento, observou-se que tais lesões ocorriam principalmente após acidentes automotivos, em especial com sistemas que utilizavam “cinto de dois pontos”. Esse fato levou a lesão a ser conhecida como “fratura do cinto de segurança”. A fratura de Chance, ou fratura de cinto de segurança, como descrita inicialmente, afeta apenas os elementos ósseos vertebrais posteriores. Portanto, geralmente é tratada de modo conservador com órtese plástica ou gessada em hiperextensão.

No entanto, quando há ruptura do complexo da banda de tensão posterior, como observado no presente caso, há necessidade de intervenção cirúrgica, devido à impossibilidade de cicatrização ligamentar adequada, fato que leva à instabilidade mecânica.^6^ Essas lesões são geralmente descritas como fratura de Chance com envolvimento ligamentar.

As fraturas de Chance são causadas por mecanismos de alta energia e raramente são descritas durante a prática de esportes. O único caso relatado de fratura de Chance durante atividade esportiva foi de queda de altura aproximada de 3,5m, durante prática de *snowboard*.^7^ Porém, há alguns relatos de fraturas toracolombar (AO tipo A) durante esportes como rúgbi, futebol, basquetebol, luta olímpica, esportes de inverno e mergulho.^3-5^

O exame físico detalhado pode revelar dor dorsal, hematoma ou intervalo doloroso na linha média dorsal. Esse último é bastante sugestivo de lesão ligamentar posterior. Neste relato, tais lesões não foram reconhecidas durante a abordagem inicial feita ainda no campo de futebol. No entanto, a imobilização apropriada e o encaminhamento ao hospital para avaliação evitou lesão adicional da coluna.

O tratamento inicial de pacientes com trauma de coluna vertebral inclui imobilização apropriada do segmento afetado, uso de colete cervical e/ou prancha rígida, e avaliação ortopédica imediata.^8^ A detecção de alargamento do espaço interespinhoso no raio X ou na tomografia computadorizada sugere lesões tipo distração-flexão. Tais fraturas também podem apresentar compressão mínima da porção anterior do corpo vertebral.^6^ Quando há suspeita de ruptura ligamentar posterior, a ressonância magnética permite melhor identificação da lesão.^7^ Além disso, a ressonância magnética possibilita informação importante sobre outras estruturas moles, como discos intervertebrais, medula espinal e raízes, especialmente quando há *déficit* neurológico.

O tratamento é definido de acordo com a presença de instabilidade mecânica ou défice neurológico.^6-8^ Lesões puramente ósseas, como descritas por Chance, podem ser tratadas com órtese em hiperextensão e apresentar bons resultados clínicos.^6^ A presença de lesão ligamentar completa, como observado nesse relato, é uma indicação para estabilização do coluna vertebral, de modo a prevenir outras instabilidades.^6,7,9^ Como os elementos anteriores são geralmente intactos, não há necessidade de reconstrução da coluna anterior.^6^

No presente relato, a instrumentação foi realizada com fixador interno AO e parafusos tipo Schanz (Synthes, Estados Unidos). O sistema possibilita braço de alavanca, que facilita a redução da fratura e a restauração do contorno sagital da coluna vertebral.^10^ A fusão adequada da coluna vertebral é esperada de 3 a 6 meses após a cirurgia, e pode ser confirmada por tomografia computadorizada. Em nosso estudo, esse exame foi realizado 6 meses após a cirurgia e mostrou fusão sólida em T11-T12. Nesse momento, a reabilitação foi intensificada, não havendo mais restrições para prática esportiva.

## CONCLUSÃO

O presente relato alerta sobre a ocorrência de lesões graves da coluna durante a prática de futebol e a importância de assistência inicial adequada ainda no campo do jogo, de modo a evitar lesões neurológicas iatrogênicas. A presença de lesão completa do complexo ligamentar posterior deve ser considerada mecanicamente instável e elegível ao tratamento cirúrgico.
